# Antagonistic interaction between posaconazole and olorofim in a murine model of invasive pulmonary aspergillosis

**DOI:** 10.1093/jac/dkaf355

**Published:** 2025-09-24

**Authors:** Christopher A Darlow, Nicola Farrington, Anne-Grete Märtson, Adam Johnson, Laura McEntee, Iona Horner, Adam Stevenson, Ana Jimenez-Valverde, Jennifer Unsworth, Shampa Das, William Hope

**Affiliations:** Antimicrobial Therapeutics and Pharmacodynamics, University of Liverpool, Liverpool, UK; Antimicrobial Therapeutics and Pharmacodynamics, University of Liverpool, Liverpool, UK; Antimicrobial Therapeutics and Pharmacodynamics, University of Liverpool, Liverpool, UK; Leiden Academic Centre for Drug Research, Leiden University, Leiden, the Netherlands; Antimicrobial Therapeutics and Pharmacodynamics, University of Liverpool, Liverpool, UK; Antimicrobial Therapeutics and Pharmacodynamics, University of Liverpool, Liverpool, UK; Antimicrobial Therapeutics and Pharmacodynamics, University of Liverpool, Liverpool, UK; Antimicrobial Therapeutics and Pharmacodynamics, University of Liverpool, Liverpool, UK; Antimicrobial Therapeutics and Pharmacodynamics, University of Liverpool, Liverpool, UK; Antimicrobial Therapeutics and Pharmacodynamics, University of Liverpool, Liverpool, UK; Antimicrobial Therapeutics and Pharmacodynamics, University of Liverpool, Liverpool, UK; Antimicrobial Therapeutics and Pharmacodynamics, University of Liverpool, Liverpool, UK

## Abstract

**Background:**

Olorofim is a new antifungal agent with a novel mechanism of action with *in vitro* activity against *Aspergillus fumigatus* and other clinically important moulds. As antifungal combinations are of interest to extend the spectrum of coverage and improve antifungal activity, we investigated the pharmacodynamics of olorofim in combination with posaconazole.

**Methods:**

Using galactomannan as a pharmacodynamic endpoint, olorofim and posaconazole were assessed alone and in combination in a neutropenic murine model of pulmonary aspergillosis using wild-type and triazole-resistant strains of *A. fumigatus*. Pharmacokinetic and pharmacodynamic data were fitted to a pharmacodynamic interaction model. Monte Carlo simulations of human-like regimens of both agents alone and in combination were performed to extrapolate to clinical settings.

**Results:**

With the triazole-susceptible isolate, both monotherapy arms suppressed galactomannan, but suppression with the combination was less than expected from the monotherapy arms. With the triazole-resistant isolate, monotherapy produced galactomannan suppression with olorofim but not posaconazole; the combination arm produced less suppression than olorofim alone. The interaction model revealed antagonistic pharmacodynamic interaction parameter values. Extrapolations to human pharmacokinetics predicted that combination therapy would still have a net beneficial effect in *A. fumigatus* infections, albeit with reduced efficacy in infections with triazole-resistant isolates.

**Conclusions:**

Posaconazole reduces the effect of olorofim *in vivo*. A combination of olorofim and a mould-active triazole is likely efficacious in wild-type infections but may be suboptimal in triazole resistance infections where there is minimal contribution of the mould-active triazole to antifungal activity and the triazole antagonises olorofim to produce a submaximal effect.

## Introduction

*Aspergillus* spp. are a persistent cause of excessive morbidity and mortality in immunocompromised patients.^1,2^ The World Health Organization has recently published a list of fungal priority pathogens, which includes *Aspergillus fumigatus*, *Fusarium* spp., *Lomentospora prolificans* and *Scedosporium* spp.^3^ Triazole resistance in *Aspergillus* spp. is an increasing concern and is associated with poor clinical outcomes.^4–7^ Since triazoles are first-line agents for the prevention and treatment of invasive aspergillosis,^8,9^ new antifungal agents are required to provide options for safe and effective antifungal therapy.

Olorofim is the first member of the novel orotomide class of antifungal agents.^10,11^ It inhibits fungal *de novo* pyrimidine biosynthesis via reversible inhibition of the dihydroorotate dehydrogenase (DHODH) enzyme.^11^ Disrupting pyrimidine biosynthesis leads to impaired nucleic acid synthesis and inhibition of fungal growth.^11,12^ Olorofim has potent *in vitro* activity against medically important hyaline moulds (e.g. *Aspergillus* spp., *Lomentospora prolificans*, *Scedosporium* spp., *Penicillium* spp. and some *Fusarium* spp.), thermally dimorphic fungi (e.g. *Talaromyces marneffei*) and the endemic fungi (*Coccidioides* spp., *Histoplasma capsulatum* and *Blastomyces dermatitidis*).^11,13–20^ Importantly, olorofim retains activity against triazole-resistant moulds with no microbiological or pharmacodynamic evidence of cross-resistance.^11,13,21^ Further, it is orally bioavailable, rendering it a potential alternative to oral triazole treatment.^22^ In a recently published Phase 2b trial, it demonstrated efficacy and tolerability in the treatment of difficult-to-treat fungal infections.^23^

There has been a long-standing interest in combining antifungal agents to increase the spectrum of activity and potentially achieve improved antifungal activity and clinical effect, particularly in the context of refractory disease.^24,25^ Recently, it has been shown that olorofim and voriconazole are antagonistic in static *in vitro* settings, but this phenomenon is genus-, species-, strain- and azole-specific.^26,27^ This is potentially problematic as the triazole posaconazole is used frequently for fungal prophylaxis in immunocompromised individuals, such as those treated for haematological malignancies.^8,9^ Here, we examined the potential pharmacodynamic interaction of posaconazole and olorofim in a well-characterized experimental model of invasive pulmonary aspergillosis.

## Materials and methods

### Challenge strains

*A. fumigatus* NIH4215 (triazole wild type) and *A. fumigatus* 11628 (Cyp51A G138C mutant) were used in these studies, with posaconazole MIC values of 0.125 and 0.5 mg/L, respectively. Olorofim MIC values were 0.03 mg/L for both isolates, as previously described.^22^

For murine studies, NIH4215 and 11628 were grown on Sabouraud dextrose agar containing chloramphenicol (SABC) (Sigma-Aldrich, Gillingham, UK). Flasks were incubated at 37°C for at least 5 days. To harvest conidia, the surface of the flask was gently irrigated with phosphate-buffered saline (PBS) pH 7.2 (Thermo Fisher Scientific, Loughborough, UK) with 0.05% Tween 80 (Sigma-Aldrich, Gillingham, UK), and the resultant mixture centrifuged at 575 × ***g*** for 10 min. The pellet was resuspended in PBS with 0.05% Tween 80. Conidia were washed through sterile gauze, and the suspension was progressively diluted to achieve a density of 1 × 10^7^ cfu/mL. The suspension was used immediately for inoculation and was confirmed with quantitative counts.

### Antifungal agents

For LC-MS/MS, *in vitro* and *in vivo* testing, olorofim powder was supplied by F2G Ltd (Cheshire, UK) and stored at −20°C. For *in vitro* testing, olorofim was dissolved in dimethyl sulfoxide (DMSO) as necessary. For *in vivo* murine studies, olorofim was dissolved in 5% DMSO (Thermo Fisher Scientific, Loughborough, UK) and vortexed vigorously before being supplemented with 10% polyethylene glycol (PEG) 400 (Sigma-Aldrich, Gillingham, UK). For the vehicle, 15 g of (2-hydroxypropyl)-β-cyclodextrin (HPBCD) (Sigma-Aldrich, Gillingham, UK) was dissolved in 50 mL sterile water. The olorofim DMSO/PEG400 solution was mixed with HPBCD, producing a solution with the final proportion of excipients as follows: 30% HPBCD, 5% DMSO and 10% PEG. The solutions were filter-sterilized (pore size 0.22 μm) and stored at −20°C for the duration of the study. Prior to use, drug-containing vials were fully thawed and vortexed and discarded after use.

For LC-MS/MS and *in vitro* testing, posaconazole powder (Sigma-Aldrich, Gillingham, UK) was stored at 4°C and dissolved in DMSO as necessary. For *in vivo* murine studies, posaconazole oral suspension (Noxafil, MSD, Merck, UK) was stored at 4°C and diluted in 20% HPBCD (4 g HPBCD in 20 mL water) as necessary.

### Murine model of invasive pulmonary aspergillosis

Mouse studies were conducted under a UK Home Office project license approved by the Animal Welfare Ethics Review Board at the University of Liverpool. Male CD-1 mice (Charles River, Margate, UK) weighing 25–30 g at the time of experimentation were rendered neutropenic via intraperitoneal injection with cyclophosphamide (Sandoz GmbH, Germany) at 150 and 100 mg/kg at 4 days and 1 day before infection, respectively. Additionally, cortisone acetate was administered subcutaneously 1 day before infection at 250 mg/kg. This immunosuppression regimen impairs pulmonary alveolar macrophages and results in a persistent and profound neutropenia. A total of 2.5% enrofloxacin was provided in the drinking water of each mouse cage from the first day of immunosuppression to prevent secondary bacterial infection.

Prior to infection, mice were anaesthetized with 2% isoflurane/98% oxygen and infected with an inoculum of 5 × 10^5^ cfu (i.e. 50 μL/mouse of the 1 × 10^7^ cfu/mL inoculum), which was instilled into both nares (25 μL/nostril). Antifungal treatment commenced 6 h post-inoculation. Mice were treated throughout the experimental period with intravenously administered (i.v.) vehicle control, various i.v. regimens of olorofim (q8h) and orally administered (p.o.) posaconazole (q24h). The treatment duration was 72 h, meaning that the entire experimental duration was 78 h (6 h treatment delay followed by 72 h of antifungal therapy).

### Pharmacokinetic studies

For the pharmacokinetic (PK) studies, agents were studied in combination. Olorofim was administered i.v. q8h, and posaconazole was administered via oral gavage (p.o.) q24h. The following agent combination regimens were studied: olorofim 4 mg/kg q8h + posaconazole 5 mg/kg q24h, olorofim 8 mg/kg q8h + posaconazole 10 mg/kg q24h and olorofim 15 mg/kg q8h + posaconazole 15 mg/kg q24h.

Blood samples (approximately 1 mL) were collected for both olorofim and posaconazole at predefined times of 0 (pre-dose), 0.5, 1, 2, 4 and 8 h post-dose in the first dosing interval for both compounds and the third and seventh dosing intervals for posaconazole and olorofim, respectively. Whole blood was collected by terminal cardiac puncture following anaesthesia induced with 5% isoflurane/95% oxygen. Blood samples were collected with heparinized syringes before being placed in Eppendorf tubes, centrifuged at 13 000 × ***g*** for 2 min, the plasma supernatant was removed, and samples were stored at −80°C.

Samples were analyzed for olorofim and posaconazole concentrations as follows. Olorofim and posaconazole were extracted from mouse plasma: [^2^H_5_]-posaconazole (Alsachim, France) was used as the internal standard and dissolved in acetonitrile (Thermo Fisher Scientific, UK) to achieve a final concentration of 5 mg/L. A total of 150 μL was added to a 96-well Phenomenex protein precipitation plate. A total of 50 μL of mouse plasma samples, blanks, calibrators (ranging from 0.025–25 mg/L) and quality control samples (0.075, 0.75, 7.5 and 12.5 mg/L) were aliquoted and mixed with the internal standard on an orbital shaker for 5 min. The liquid was drawn through the protein precipitation plate using a positive pressure manifold. The samples were evaporated under nitrogen (40 L/min) for 30 min followed by reconstitution in 150 μL of 80:20 water:ACN (Thermo Fisher Scientific, UK) and 0.1% formic acid (Sigma-Aldrich, UK). The plate was sealed and placed onto an orbital shaker for 10 min prior to analysis by LC-MS/MS. LC-MS/MS analysis was performed using an Agilent 1290 Infinity HPLC coupled to an Agilent 6420 triple quadrupole mass spectrometer fitted with an electrospray source. The LC-MS system was controlled using Agilent MassHunter Data Acquisition software (Ver B.06.00). Analytes were injected (5 μL) onto Phenomenex Kinetex EVO C18 100 Å (2.1 mm × 50 mm, 2.6 µm, 40°C) and separated over a 3.5 min gradient using a mixture of Solvents A and B. Solvent A was LC-MS grade water with 0.1% (v/v) formic acid. Solvent B was LC-MS grade acetonitrile with 0.1% (v/v) formic acid. Separations were performed by applying a linear gradient of 5%–95% Solvent B over 2.5 mins at 0.6 mL/min followed by an equilibration step (1 min at 5% Solvent B). The mass spectrometer was operated in positive ion mode using a multiple reaction monitoring method. Following an optimization process, the following mass transitions and collision energies were used for analysis: 499.1 > 198.0 (Ce 20 ev), 701.2 > 126.9 (Ce 60 ev) and 706.2 > 126.9 (Ce 60 eV). The mass spectrometer conditions were as follows: capillary voltage of 3.5 kV; fragmentary voltage of 100 V; source gas temperature of 350°C; and gas flow of 11 L/min. The data were processed using Agilent MassHunter Quantitative Analysis (Ver B.05.02).

### Pharmacodynamic studies

The pharmacodynamic (PD) studies were performed with strains NIH4215 and 11628. The studies included the following dosing arms: olorofim 4 mg/kg q8h, olorofim 8 mg/kg q8h, posaconazole 10 mg/kg q24h, olorofim 4 mg/kg q8h + posaconazole 10 mg/kg q24h and olorofim 8 mg/kg q8h + posaconazole 10 mg/kg q24h. All studies included vehicle control arms. At planned timepoints of 30, 54 and 78 h postinfection, whole blood from multiple mice was collected by terminal cardiac puncture into non-heparinized syringes using 5% isoflurane as terminal anesthesia. Whole blood was then placed into microfuge tubes, centrifuged at 12 000 × ***g*** for 2 min, the serum supernatant was removed, and samples were stored at −20°C until galactomannan (GM) analysis was performed as the primary PD output. To measure the GM index in mouse serum samples, an immunoenzymatic sandwich ELISA kit (Platelia *Aspergillus* Kit, Bio-Rad, UK) was used according to the manufacturer’s instructions. The mean and standard error were calculated from the samples taken at each timepoint.

### Pharmacodynamic interaction modelling

All PK-PD modeling was performed with the population PK program Pmetrics.^28^ An interaction model based on constructs originally conceived by Greco^29^ was fitted to the combined PK and PD data to estimate the degree of PD interaction.

The full structural PK-PD model consisted of the following differential equations:


(1) $$\frac{dX1}{dt}=-Ka\;*X1$$

(2) $$\frac{dX2}{dt}=Ka*X1-\left(\frac{CL1\;}{V1}+KCP1\right)*X2+KPC1*X3$$

(3) $$\frac{dX3}{dt}=KCP1*X2-KPC1*X3$$

(4) $$\frac{dX4}{dt}=RATEIV(2)-\left(\frac{CL2\;}{V2}+KCP2\right)*X4+KPC2*X5$$

(5) $$\frac{dX5}{dt}=KCP2*X4-KPC2*X5$$

(6a ) $$\frac{dX6}{dt}=X6*\left(Kg*\left(1-\;\frac{X6}{POPmax}\right)-\frac{Kk*XM}{\left(1-XM\right)}\right)$$

(6b ) $$if\;X2=0\;then\;XM=\;{\left(\frac{\frac{X4}{V2}}{E50_{2}}\right)}^{H2}$$

(6c ) $$if\;X4=0\;then\;XM={\left(\frac{\frac{X2}{V1}}{E50_{1}}\right)}^{H1}$$

(6d ) $$Else:\;\frac{\left(\frac{X2}{V1}\right)}{E50_{1}\;*XMs^{H1}}+\frac{\left(\frac{X4}{V2}\right)}{E50_{2}*XMs^{H2}}+\frac{\left(\alpha *\;\frac{X2}{V1}\;*\;\frac{X4}{V2}\right)}{E50_{1}\;*\;E50_{2}\;*XM^{\frac{H1+H2}{2}}\;}=1$$

Equations (1)–(5) model the concentration profiles of posaconazole (X2) and olorofim (X4) with respect to time (t). Ka is the first-order absorption rate of posaconazole from the gut to the central compartment, RATEIV refers to the infusion rate of olorofim; CL1 and CL2 are clearance values for posaconazole and olorofim, respectively; V1 and V2 refer to the volume of distribution of each agent; KCP1, KCP2, KPC1 and KPC2 are the first-order intercompartmental rate constants connecting central and peripheral compartments.

Equations (6a)–(6d) describe the PD of both drugs and the interaction. Kg refers to the fungal growth constant; POPmax refers to the maximum GM value; and Kk refers to the fungal kill constant. XM is a conditional composite term. If X2 or X4 = 0, then equations (6b) and (6c) define these functions, where H1 and H2 refer to the Hill constants for posaconazole and olorofim, respectively. EC50_1_ and EC50_2_ refer to the concentrations of posaconazole and olorofim required to achieve 50% of maximal efficacy. In these circumstances, equation (6a) describes a standard Emax model. When X2 > 0 and X4 > 4, equation (6d) defines XM. This equation replicates Greco’s model of synergy,^29^ with α representing the interaction term for posaconazole and olorofim. Under these conditions, XM represents the composite term (E/Econ-E), where E is the measured effect of the present agents and Econ is the control response. As equation (6d) is in an unclosed form, the value of XM was determined via a Nelder–Mead algorithm.^30^ Independent models were fitted to the data for each challenge strain, given the different PD relationships.

### Simulations of effects in humans

For both fitted models, Monte Carlo simulations were conducted for 10 000 individuals in Pmetrics using the parameter covariance matrix from the fitted model and final model support points to inform the population distribution. As the model PK parameters were murine, a simulated dosing schedule was created to replicate the drug exposure of typical regimens of 400 mg q24h p.o. for posaconazole^31^ and 90 mg q12h p.o. for olorofim, replicating the regimen used in the Phase 2b study.^23,32^

## Results

### Murine model of invasive pulmonary aspergillosis

**Figure 1.**
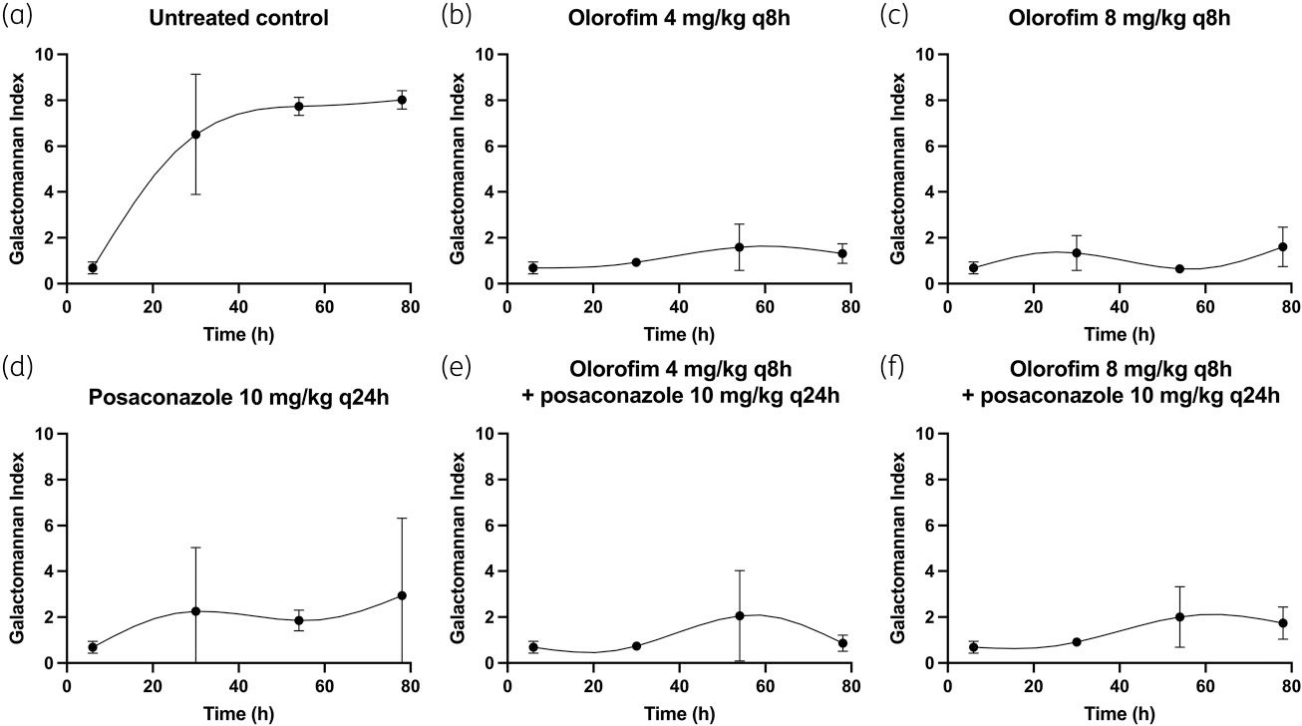
PD of olorofim and posaconazole alone and in combination in a murine model of invasive pulmonary aspergillosis with NIH4215 (posaconazole MIC = 0.125 mg/L; olorofim MIC = 0.03 mg/L) as the challenge strain. Symbols and error bars represent mean and standard error, respectively, of the GM index in the absence of antifungal agent (a), two olorofim monotherapy regimens (b and c), one posaconazole monotherapy regimen (d) and two combination regimens with both olorofim and posaconazole (e and f).

**Figure 2.**
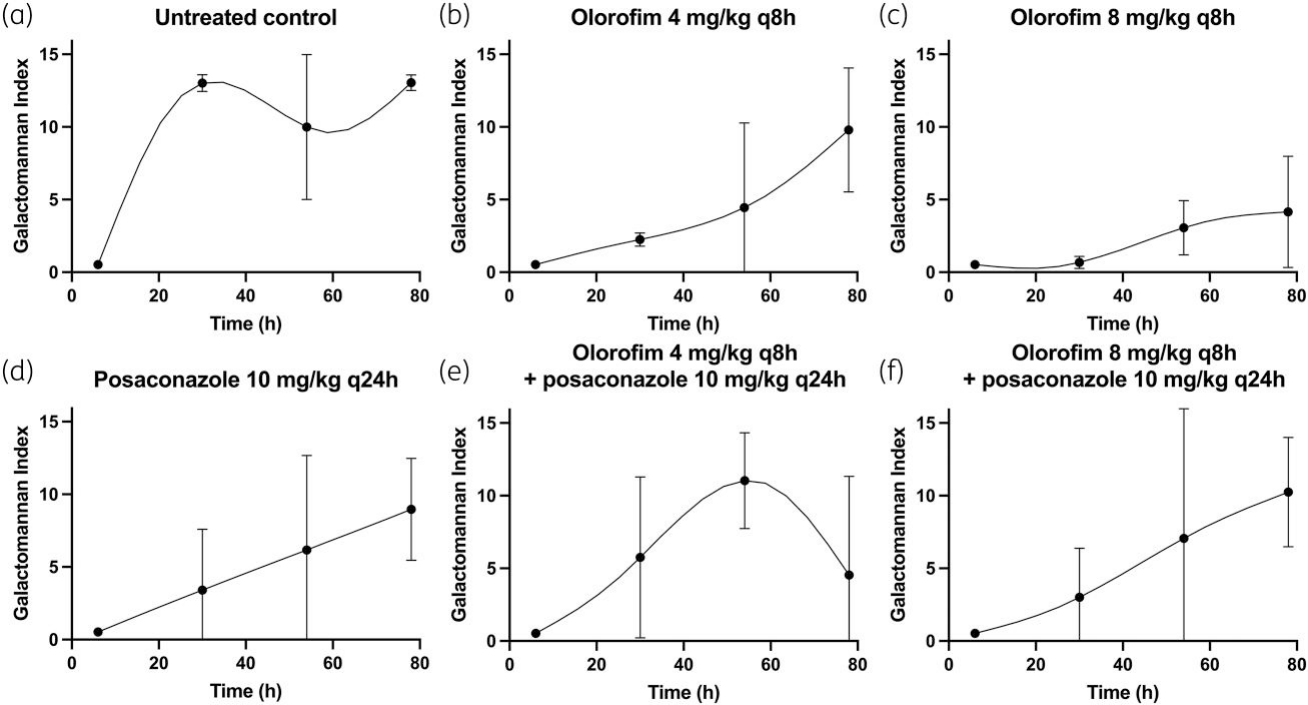
PD of olorofim and posaconazole alone and in combination in a murine model of invasive pulmonary aspergillosis with 11628 (posaconazole MIC = 0.5 mg/L; olorofim MIC = 0.03 mg/L) as the challenge strain. Symbols and error bars represent mean and standard error, respectively, of the GM index in the absence of antifungal agent (a), two olorofim monotherapy regimens (b and c), one posaconazole monotherapy regimen (d) and two combination regimens with both olorofim and posaconazole (e and f).

The murine model of invasive aspergillosis is a mimic of the rapidly progressive universally lethal disease characteristic of profoundly neutropenic patients. GM concentrations began to increase in the first 24 h postinfection and were maximal after 48 h and thereafter (Figures 1 and 2).

### Pharmacodynamic interaction studies

**Figure 3.**
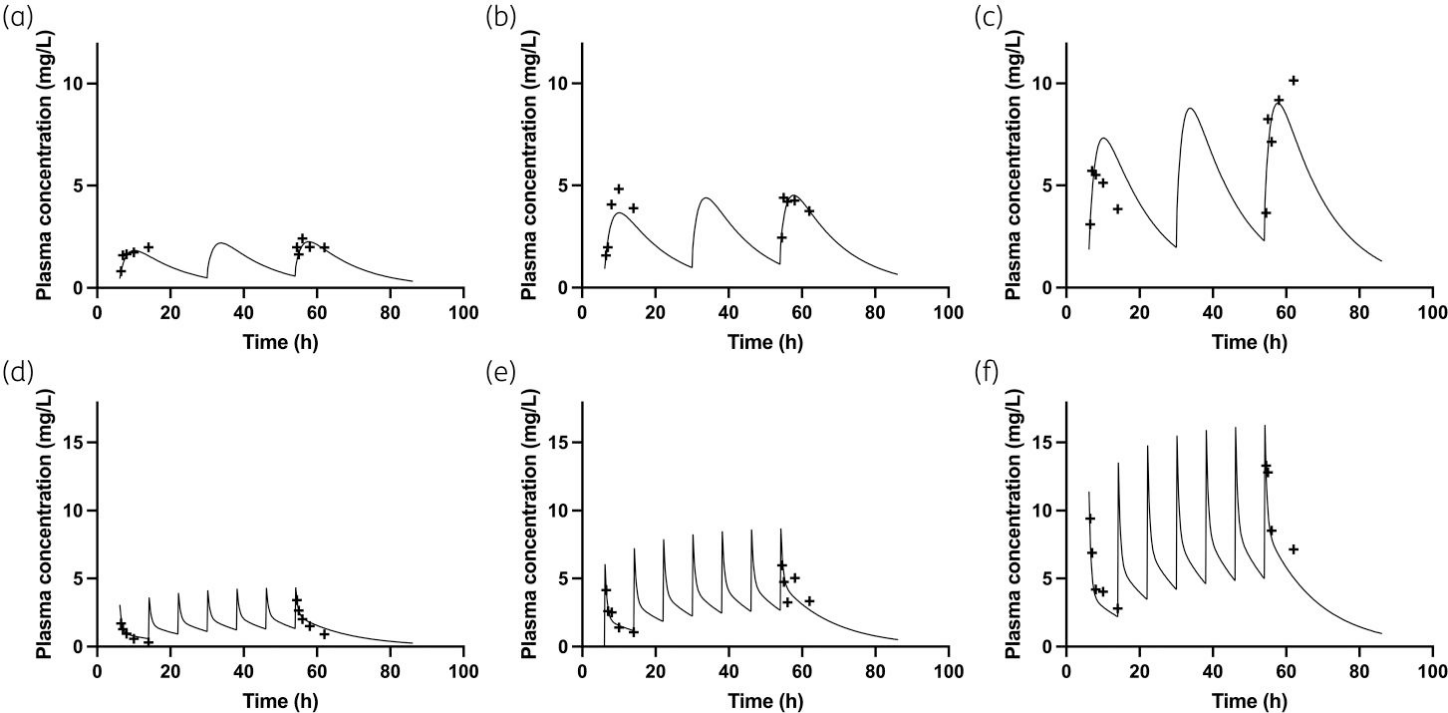
Experimental time-concentration profiles from the PK arms of the murine model for (a) posaconazole 5 mg/kg q24h p.o., (b) posaconazole 10 mg/kg q24h p.o., (c) posaconazole 15 mg/kg q24h p.o., (d) olorofim 4 mg/kg q8h i.v., (e) olorofim 8 mg/kg q8h i.v. and (f) olorofim 12 mg/kg q8h i.v. Solid lines indicate the predicted time-concentration profile using the fitted PK-PD model using the mean PK parameters. Crosses indicate measured plasma concentrations from mice receiving the relevant regimen.

For all experiments, the regimens of olorofim and posaconazole were informed from previous studies^22^ and based on knowledge of drug exposures that were both clinically relevant and resulted in on-scale readouts of GM concentrations. The PK of both olorofim and posaconazole were determined and consistent with previous studies (Figure 3).^22^

The PD of olorofim and posaconazole against the two *A. fumigatus* strains are shown in Figures 1 and 2. Olorofim monotherapy resulted in exposure-dependent reduction in GM concentrations in both NIH4215 and 11628. Similarly, posaconazole monotherapy resulted in suppression of GM concentrations in NIH4215; however, there was little effect of posaconazole against 11628, which was consistent with its elevated posaconazole MIC of 0.5 mg/L and previous experiences with this strain.^22,33^ The combination therapy arms did not achieve any additional anti-fungal effect compared with olorofim monotherapy for the NIH4215 isolate, and the effect was worse than olorofim monotherapy against 11628.

**Figure 4.**
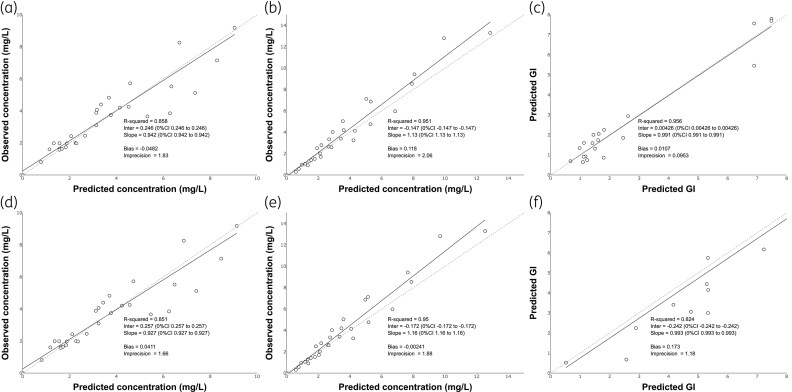
Observed-versus-predicted plots demonstrating population fit for the fitted PK-PD model using mean parameter estimates for NIH4215 (a–c) and 11628 (d–f) and for model performance by posaconazole plasma concentration (a and d), olorofim plasma concentration(b and e) and GM index (c and f). GI = GM index.

A PK-PD interaction model was fitted to the combined PK and PD dataset for each strain (Figure 4). The parameter estimates are summarized in Table 1. For both strains, the value of the credible interval for α was below zero, suggesting an antagonistic interaction between the two agents.

**Table 1.** Parameter estimates for fitted PK-PD model to the murine infection model experimental outputs for both NIH4215 and 11626

Parameter (Units)	NIH4215			11626		
	Mean	Median	95% credible interval	Mean	Median	95% credible interval
POPmax	7.49	7.54	7.38–7.70	12.97	13.15	12.29–14.00
Ka (h^−1^)	0.515	0.522	0.500–0.544	0.55	0.55	0.50–0.60
V_1_ (L)	0.016	0.016	0.016–0.017	0.016	0.016	0.015–0.017
V_2_ (L)	0.026	0.025	0.025–0.026	0.026	0.026	0.025–0.026
CL_1_ (L/h)	0.0037	0.0038	0.0035–0.004	0.0036	0.0036	0.0034–0.0040
CL_2_ (L/h)	0.0062	0.0060	0.0055–0.0065	0.0061	0.0063	0.0055–0.0065
KCP_1_ (h^−1^)	6.34	6.50	6.00–7.00	6.70	7.00	6.09–7.00
KPC_1_ (h^−1^)	3.16	3.12	3.00–3.24	3.33	3.00	3.00–4.00
KCP_2_ (h^−1^)	1.13	1.10	1.00–1.20	1.26	1.34	0.94–1.44
KPC_2_ (h^−1^)	0.534	0.550	0.50–0.60	0.56	0.567	0.94–1.45
K_g_	0.199	0.189	0.161–0.218	0.199	0.188	0.086–0.289
K_k_	0.380	0.379	0.375–0.382	0.226	0.199	0.047–0.351
E50_1_ (mg/L)	24.42	25.41	22.38–28.45	5.94	5.00	5.00–8.29
E50_2_ (mg/L)	16.21	18.81	10.83–26.80	2.83	3.31	0.184–5.25
α	−11.87	−12.09	−12.76 to −11.41	−17.50	−15.15	−30.00 to −10.18
H_1_	0.281	0.302	0.237–0.367	0.508	0.499	0.485–0.549
H_2_	0.162	0.189	0.105–0.273	0.174	0.169	0.159–0.197
IC	0.217	0.233	0.185–0.281	0.161	0.178	0.032–0.325

Parameters as defined in the Materials and methods section. IC, initial condition for GM index.

### Monte Carlo simulation and extrapolation to clinical settings

**Figure 5.**
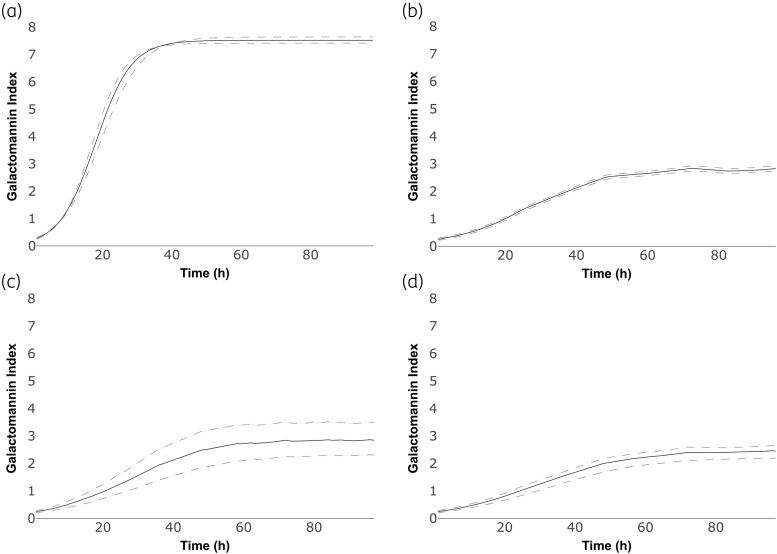
Simulation output using the NIH4215 strain PK-PD model with no treatment (a), posaconazole 400 mg q24h p.o. (b), olorofim 90 mg q12h p.o. (c) and both in combination (d). The solid line is the median GM trajectory. Dashed lines indicate 5th and 95th centiles. While the interaction is antagonistic, there is a greater effect with the combination than with either monotherapy.

**Figure 6.**
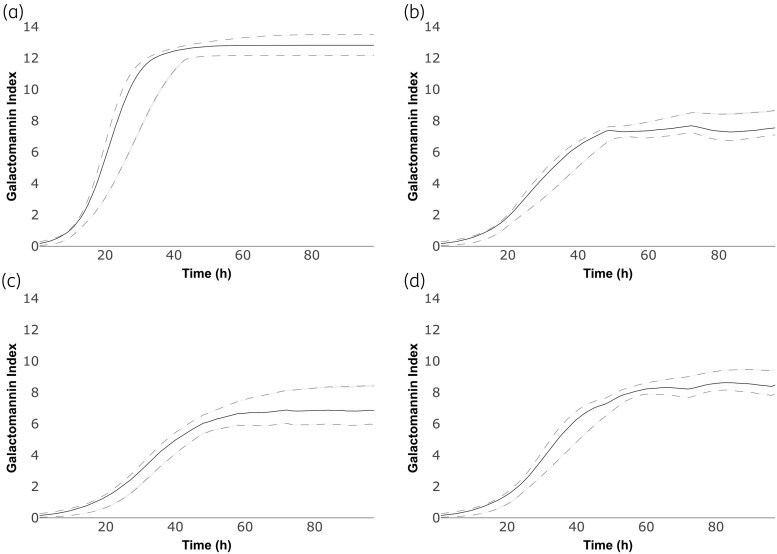
Simulation output using the 11628 strain PK-PD model with no treatment (a), posaconazole 400 mg q24h p.o. (b), olorofim 90 mg q12h p.o. (c) and both in combination (d).

Using the PK-PD models, Monte Carlo simulations were performed with a humanised PK profile of posaconazole 400 mg q24h p.o. and olorofim 90 mg q12h p.o.^31,32^ alone and in combination (Figures 5 and 6). For NIH4215 (Figure 5), both posaconazole and olorofim resulted in suppression of GM index growth over the course of the simulation. When administered in combination, the total effect was greater, but less than expected with an additive PD interaction (i.e. the effect of posaconazole alone plus the effect of olorofim alone). For 11628 (Figure 5), olorofim had a comparable effect to NIH4215 (Figures 5c and 6c) while posaconazole had little effect compared to NIH4215 (Figures 5b and 6b), which was consistent with the elevated MIC of posaconazole. In combination (Figure 6d), the effect was worse than either agent administered alone.

## Discussion

To fully understand the implications of this work and the conclusion that triazoles and olorofim are antagonistic, it is necessary to briefly overview the conceptualisation of PD interactions. If two drugs, a and b, have corresponding effects A and B, it is commonly (but imprecisely) understood that antagonism between the two drugs leads to A + B < A or B alone. However, antagonism, as conceived by the concept of Loewe additivity and further formulated in models developed by Greco *et al*.^29,34^ defines PD interactions differently. Here, drugs a and b in combination produce a total effect of (A + B + C), where C is an additional effect relating to the PD interaction of the two drugs. In Greco *et al*.’s model, α is a multiplicative factor of C and positive and negative values of α indicate synergy and antagonism, respectively. An α value of zero removes C entirely and indicates an additive interaction.

In antagonism (i.e. with negative α and C values), the overall effect of combination therapy of drugs a and b therefore depends on the magnitude of A and B in relation to C. If A and B are greater in magnitude than C, then (A + B − C) > A or B alone. Conversely, if at least one of A and B is smaller in magnitude than C, then (A + B − C) < A or B alone. That is to say that an antagonistic relationship between two drugs can still produce a combined effect that is greater than either monotherapy alone.

In this instance, both posaconazole and olorofim have significant effects against *A. fumigatus* isolate NIH4215 as monotherapy and, despite the negative α (i.e. negative C), produce an effect in combination greater than either monotherapy due to the magnitude of effect of both agents for this strain. In contrast, *A. fumigatus* isolate 11628 is relatively non-susceptible to posaconazole, and the lower effect of this agent (in comparison to NIH4215) means that the antagonistic combination therapy produces an effect in combination that is less than or equal to either agent alone.

While our work did not provide insight into the exact mechanism of the antagonism between triazoles and olorofim, previous work has suggested a mechanism.^26^ It is known that the mechanism of action of olorofim is via disruption of pyrimidine biosynthesis, preventing DNA/RNA synthesis and fungal growth.^11,26^ Accordingly, supplementation of exogenous pyrimidine negates the activity of olorofim.^11,26^ Van Rhijn *et al*.^26^ demonstrated that the antagonism between itraconazole and olorofim seemed to be mediated by itraconazole-induced upregulation of the pyrimidine biosynthesis pathway, thus reducing the effect of olorofim by the same principle.

In a clinical context, the *in vivo* antagonism observed in this model between olorofim and posaconazole results in an overall net beneficial effect when used to treat *A. fumigatus* strains that are susceptible to both agents, but a reduced overall effect when used for a posaconazole-resistant strain. However, distinguishing these two contexts of invasive aspergillosis requires confirmation of the diagnosis through culture and, subsequently, antifungal susceptibility testing with posaconazole. Treating patients empirically with olorofim and posaconazole in combination (either as a conscious combination treatment choice or with continuation of pre-existing posaconazole prophylaxis) may risk a net negative therapeutic effect for a triazole-resistant *Aspergillus* species compared to olorofim monotherapy. The existence of this effect in patients needs to be further validated. However, such a signal has not been detected in the limited clinical data currently available.^35^ It should also be noted that prior reported preclinical antagonism between antifungal combinations has not translated to clinical significance.^36^ For example, it had been demonstrated that amphotericin B and flucytosine were antagonistic when used against *Cryptococcus neoformans* in preclinical models.^37–39^ However, this pharmacologically defined antagonism never materialised as a significant effect in clinical settings.^40^ Similarly, concerns about azole-polyene antagonism in both *in vitro* and *in vivo* models of aspergillosis have not materialised in humans.^41,42^ Thus, it may well prove to be the case that the pharmacological antagonism observed in the murine model between posaconazole and olorofim does not translate to a clinically significant effect.

Nevertheless, it seems prudent to avoid unnecessary combination treatment with olorofim and posaconazole wherever possible, especially in cases of suspected or confirmed triazole resistance, or in cases where triazole drug exposure is likely to be subtherapeutic, particularly if an alternative combination partner, e.g. an echinocandin or polyene, is available. Further, although this study was limited to posaconazole, given the observed *in vitro* antagonistic interaction between olorofim and both voriconazole (in a strain-dependent manner) and isavuconazole (but not fluconazole),^27^ the antagonistic effect seen in our work is likely to be similar to these mould-active agents too. Additional experiments would be needed with these agents to replicate this interaction effect *in vivo*. Absent that work, it seems reasonable to extend the aforementioned caution to coadministration with these mould-active triazole agents when treating invasive aspergillosis.

There are limitations to our work. First, this experimental work used serum galactomannan as a quantitative proxy for fungal burden of disease. This is largely because quantitative fungal culture in animal models is unreliable compared to galactomannan measurement, often underestimating actual fungal burden.^43^ Although galactomannan has a track record of being used in murine models of *Aspergillus* infections to assess the pharmacodynamics of antifungals,^22,44,45^ the indirect measure of fungal burden in this work should be acknowledged.

Secondly, we simulated the maximal human-like drug exposures in the *in vivo* model and assumed that the antagonistic interaction remains constant across all drug concentrations in the *in silico* simulations using standard clinical doses. It is computationally impossible to do otherwise, but this does not account for the possibility of a concentration-dependent pharmacodynamic interaction effect that is suggested by the work of both Pinder *et al*. and Laloum *et al*.^27,46^

Finally, this work examined in detail only the interaction of olorofim and posaconazole with *A. fumigatus*. We cannot comment on the potential pharmacodynamic interaction of these two agents (or other mould-active azoles and mould fungi) when used in combination for the treatment of other fungi, including other species of *Aspergillus*. Of note, the available clinical data documents successful use of olorofim in combination with both fluconazole and the mould-active triazoles in the treatment of coccidioidomycosis.^23^

The complexity of fungal biology and therapeutics underlines the need for the comprehensive examination of combination antifungal regimens for the treatment of invasive fungal infections. A progressive understanding of the mechanism of interaction, experimental models that are highly predictive of clinical therapeutics and advances in characterization of pharmacodynamic interactions suggest that our results are likely to be clinically relevant. Our approach provides a way that combinations of antifungal agents can be examined and potential benefits and liabilities can be assessed with suitable mitigation if required.

## Transparency declarations

W.H. holds or has recently held research grants with UKRI, F2G and Global Antibiotic Research and Development Partnership (GARDP). He is (or has recently been) a consultant for F2G and GSK. He was a member of the Specialist Advisory Committee for GARDP (2020–23), a member of the British Society for Antimicrobial Chemotherapy (BSAC) Breakpoint Committee (2020–23), a member of Health Technology Appraisal (HTA) Prioritisation Committee for hospital care and was the Specialty National Co-lead for Infection for the National Institute of Health Research (NIHR) (2020–24). He has participated on an advisory board for Basilea. All other authors declare no competing interests.
